# Mucosal-adapted bacteriophages as a preventive strategy for a lethal *Pseudomonas aeruginosa* challenge in mice

**DOI:** 10.1038/s42003-024-07269-0

**Published:** 2025-01-06

**Authors:** Luiz Felipe Leomil Coelho, Mateus de Souza Terceti, Sergio Pereira Lima Neto, Raíne Piva Amaral, Ana Luisa Cauvila dos Santos, William Permagnani Gozzi, Bianca Andrade de Carvalho, Gustavo Aparecido da Cunha, Maria Fernanda Romboli Durante, Lais Sanchietta, Giovana Soares Marangoni, Matheus Luca Carotta Gabriel, Luiz Cosme Cotta Malaquias, Eliana Leonor Hurtado Celis, Giovanna de Souza Apolinário, João Pessoa Araujo Junior, Carine Ervolino de Oliveira, Victoria Fulgencio Queiroz, Gabriel Magno de Freitas Almeida

**Affiliations:** 1 Vaccine Laboratory, Department of Microbiology and Immunology, Institute of Biomedical Sciences, Federal University of Alfenas, Alfenas, Brazil; 2 Institute of biotechnology, Sao Paulo State University - UNESP, Sao Paulo, Brazil; 3 Institute of Biomedical Sciences, Department of Pathology and Parasitology, Federal University of Alfenas, Alfenas, Brazil; 4 Federal University of Minas Gerais, Institute of Biological Sciences, Department of Microbiology, Belo Horizonte, Minas Gerais Brazil; 5 The Norwegian College of Fishery Science, Faculty of Biosciences, Fisheries and Economics, UiT—The Arctic University of Norway, Tromsø, Norway

**Keywords:** Bacteriophages, Phage biology

## Abstract

*Pseudomonas aeruginosa* is an emergent threat due to the antimicrobial resistance crisis. Bacteriophages (phages) are promising agents for phage therapy approaches against *P. aeruginosa*. It has been proposed that metazoans harbor phages on their mucosal surfaces, and this could be exploited for the rational design of prophylactic phage therapy. The goal of this study was to evaluate the potential of phage-mucus interaction to prevent infections caused by *P. aeruginosa*. We isolated two phages capable of infecting *P. aeruginosa*. Both are similar in morphology and closely related genetically. However, phage VAC3 is more efficient in replicating in mucin-exposed *P. aeruginosa* in vitro and is preferentially held in the respiratory tract of C57BL/6 mice. Pre-treatment with VAC3 phage protects mice from a lethal dose of *P. aeruginosa* while VAC1 does not. This shows that phages adapted to mucosal conditions have potential to be applied as prophylactic measures against an ESKAPE pathogen.

## Abstract (web summary)

Phage VAC3 adaptations to the mucosal environment (improved replication in mucin-treated hosts and retention in the respiratory tract) are mechanisms that can exploited as prophylactic measure to protect mice from a lethal dose of *P. aeruginosa*.

## Introduction

Antimicrobial resistance (AMR) is an emerging public health problem. It is estimated that 4.95 million deaths in 2019 could be associated with bacterial AMR^1^ and that by 2050 around 10 million people will die annually from infections caused by multidrug-resistant (MDR), extensively drug-resistant (XDR), or pandrug-resistant (PDR) bacteria^2^. Therefore, innovative strategies to control the spread of MDR/XDR/PDR microorganisms are needed. Phage therapy, the use of bacteriophages (phages) as antibacterial agents, is considered a potential therapeutic substitution or adjuvant therapy to antibiotics and currently holds immense relevance for scientific and technological development in controlling MDR/XDR/PDR infections^3^. Developed in the early 20th century, phage therapy was widespread until a shift towards antibiotic therapy took place in the Western World^4^.

The increase in bacterial resistance has renewed the interest in the therapeutic potential of bacteriophages. There are reports of successful cases in humans where phage therapy has been promising in eliminating infections caused by MDR microorganisms^5–8^, such as *Acinetobacter baumannii, Mycobacterium abscessus*, and *Pseudomonas aeruginosa*. Infections caused by resistant *P. aeruginosa* are considered extremely serious and one of the main contemporary problems regarding the AMR crisis^9–11^, especially in cystic fibrosis (CF) patients. Bacteriophages that infect the *Pseudomonas* genus were first discovered in the mid-20th century, and several species have been isolated and described to date^7,12,13^. *P. aeruginosa* was the subject of one of the largest modern phage therapy clinical trials ever conducted (Phagoburn)^14^. A retrospective analysis of 100 consecutive cases of modern personalized phage therapy in Belgium revealed that 45 cases had the involvement of *P. aeruginosa*, and that phage therapy led to clinical improvement in 37 of these cases^15^. Traditional phage therapy is based on curing an already established disease. The benefits of such an approach are indisputable. However, phage therapy also has the potential for being used as prophylaxis, improving its benefits in reducing mortality and morbidity from bacterial infections. The prophylactic use of bacteriophages has been recorded in laboratory conditions and on a large scale in human populations^16–18^, although a clear mechanism explaining their preventive efficacy had not yet been described until recently. Recent research using two different in vivo models based on fish pathogens hints that interactions between phages and mucosal layers could be the explanation behind the prophylactic potential of phages^19,20^. The connection between mucosal adherence and phage-based prophylaxis of bacterial infections has also been shown in a mice model using *Escherichia coli* and phage øPNJ-6^21^.

Despite these pioneering studies, little attention has been given to the isolation and characterization of mucosal-adapted phages. A factor to consider in studying phages is their interaction with the environment, particularly with mucosal surfaces in various animals. These surfaces are habitats for many microorganisms, including bacteriophages and their hosts. Mucus is an essential component of the innate immune system and serves as a selective barrier between organisms and their environments^19,22–25^. It is known that many organisms host bacteriophages on their mucosal surfaces in a symbiotic relationship, where phages provide protection to hosts against bacterial infections, and the organism provides a means for phages to infect bacterial hosts^24^. Therefore, the bacteriophage adherence to mucus (BAM) model^24^ proposes that phages concentrate on mucosal surfaces through weak interactions with mucins, creating an immunity not derived from the host against bacterial invaders during the mucous colonization process. This phage-mucus interaction is primarily carried out by Ig-like phage structural proteins, which have been identified in about 25% of the species studied^24^. Recently, BAM model dynamics have been shown to apply to a group of four *P. aeruginosa* phages in vitro. Three of the phages had mucin-binding properties, while two were able to replicate more efficiently in mucin-exposed *P. aeruginosa*^26^. This shows that likely phage-mucin interactions can play a role in favoring phage-mediated prophylaxis in *P. aeruginosa* cases, but no in vivo data was presented.

In this study, we isolated two phages from Brazilian environmental samples and showed that one of them replicates better in mucin-exposed hosts, persists in the respiratory tract of mice, and can be used as prophylaxis against *P. aeruginosa* infections in vivo. This shows that even phages infecting the same host might possess distinct lifestyles concerning mucosal interactions, and this can be exploited for rational prophylactic approaches against clinically relevant pathogens. Phage therapy based on mucosal-adapted phages holds the potential for the protection of mucosal membranes against infections. The administration of these phages preventively in susceptible patients could prevent bacterial colonization in these anatomical sites, as these phages remain in the body for a longer time after administration. Therefore, it is a potential relevance of phage-mucus interactions for designing phage treatments.

## Results

### VAC1 and VAC3 are two phages that infect *P. aeruginosa*

**Fig. 1:**
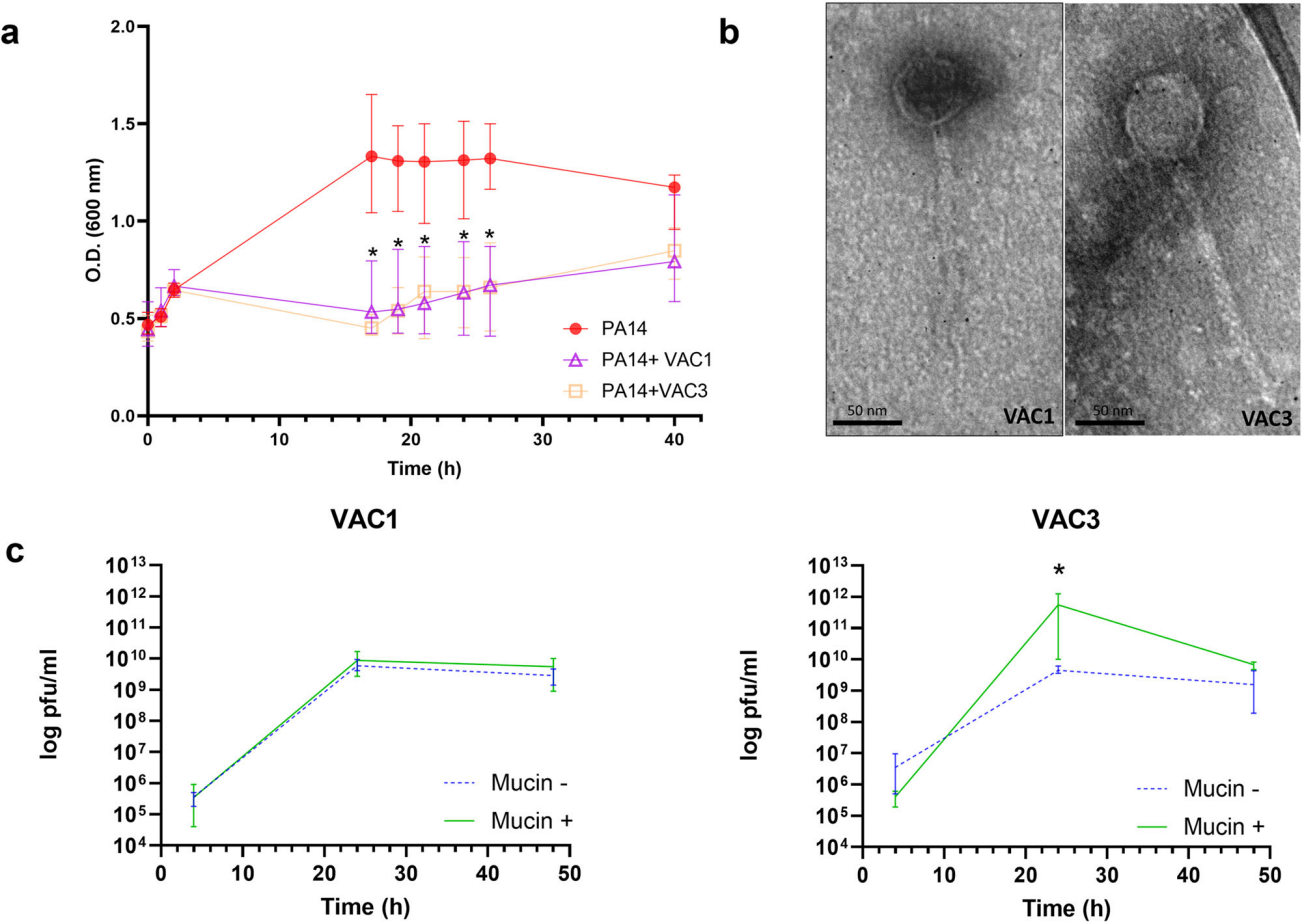
In vitro characterization of phages VAC1 and VAC3. **a** Activity of the phages VAC1 and VAC3 against *P. aeruginosa* PA14. **b** Transmission electron micrograph of phages VAC1 and VAC3. Scale bar: 50 nm. **c** VAC1 and VAC3 titers in LB cultures supplemented with 0.1% (w/v) purified porcine mucin at the time of infection. Experiments were performed in three replicates, and the data is represented as the mean ± standard deviation range in a and in c. The phage concentration at the time of infection in (**c**) was 4 × 10^4^ PFU/ml for all cultures. *p > 0.05 using unpaired *t*-student test for each individual time point.

A bioprospection effort aimed at finding phages capable of infecting *P. aeruginosa* PA14 was made using environmental water samples collected from two southeastern cities from Minas Gerais and São Paulo states. Visible round plaques in double-layer agar overlay plates appeared following the enrichment cultures of two of our samples. These isolates were plaque-purified and further propagated. These two new phages, named *P. aeruginosa* phage VAC1 and *P.* aeruginosa phage VAC3, are able to reduce the proliferation of *P. aeruginosa* PA14 in planktonic cultures. A significant reduction in *P. aeruginosa* load was seen from 18 to 40 h post infection (*p* = 0.0002) (Fig. 1a). Transmission electron micrograph (TEM) showed that phages VAC1 and VAC3 have similar-tailed morphologies since both present an icosahedral head and a long tail. TEM images showed that phage VAC1 had a head of 58.82 ± 1.7 nm in diameter and a tail with a length of 183 ± 3.03 nm. Phage VAC3 had a head of 54.75 ± 6.66 nm in diameter and a tail of 202.23 ± 27.62 nm (Fig. 1b). At least 10 viral particles were measured to calculate these dimensions. To verify the influence of mucin in the replication of phages VAC1 and VAC3, *P. aeruginosa* PA14 strain was infected with either phage in the presence of 0.1% mucin and phage progeny was measured by titration over time. VAC1 titers in the presence and absence of mucin remained similar, while VAC3 titer at 24 h of infection was almost 3 logarithmic orders higher when the infection was done in the presence of mucin (*p* = 0.0286) (Fig. 1c). Both phages are resistant to different pH and temperatures, with VAC1 likely being more affected by temperature increase (Supplementary Fig. 1).

### Phages VAC1 and VAC3 are genetically similar but not identical

**Fig. 2:**
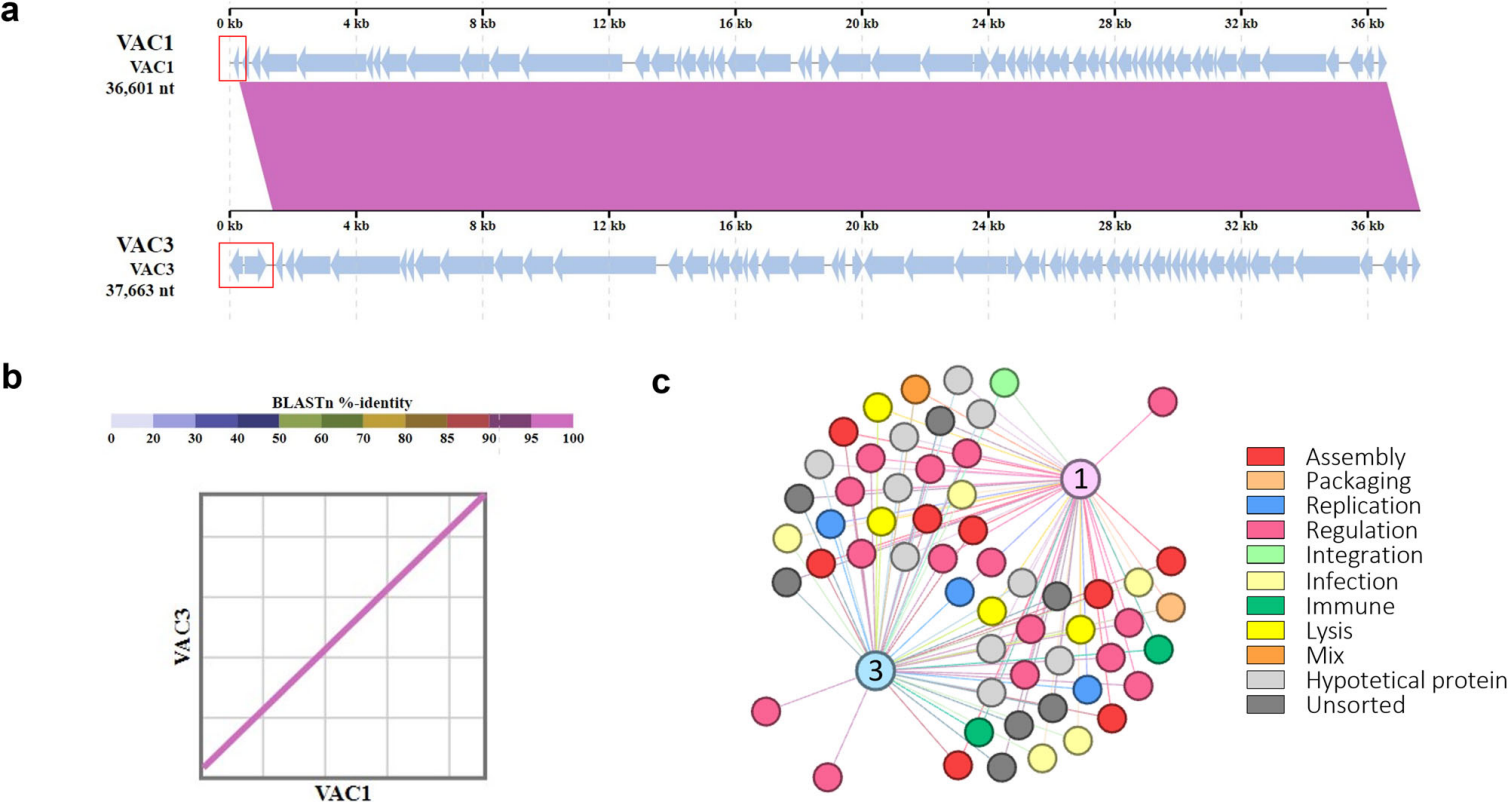
Genome comparison of phages VAC1 and VAC3. **a** Genomic synteny analysis. Comparison of genome composition and organization. The schematic whole-genome-alignment diagram was obtained on the PhageScope platform using the sequence alignment analysis. Red boxes indicate asyntenic regions, and blue arrows indicate the position and orientation of the ORFs. **b** Dot plot showing genetic collinearity and identity between covered regions. **c** A network graph illustrating the components of the pangenome. The pangenome comprises a total of 113 genes grouped in 57 clusters, of which 54 are shared, constituting the core genome. Larger nodes represent the bacteriophages VAC1 (1) and VAC3 (3). Smaller nodes represent individual clusters, with each color indicating its functional category in the final annotation. The graph was generated using a force-based algorithm.

The phages used in this study have circular dsDNA genomes of 36601 (VAC1) and 37663 (VAC3) base pairs (bp) in size (Fig. 2a). Genome comparison of VAC1 and VAC3 phages demonstrate a conserved synteny with a high degree of identity (Fig. 2b), except for a unique region of 1062 bp present in the 5′ region of VAC3 phage. Phylogenetic analysis based on amino acid sequences of homologous major capsid proteins of VAC1 and VAC3 genes showed that they are highly similar and belong to the same lineage (Supplementary Fig. 2). VAC1 genome contains 56 predicted ORFs and a 64.1% G + C content while VAC3 genome contains 57 ORFs as well as a 64.2% G + C content (Supplementary Fig. 3 and Supplementary Data Set 1). The pangenome analysis identified that 55 ORFs are shared between the two phages (Fig. 2c). The annotation indicates that the only gene exclusive to VAC1 is an intermediate transcription factor (VAC1_ORF1) while the two genes exclusive to VAC3 codes for a LysR substrate-binding domain-containing protein (VAC3_ORF1) and a nucleoside-specific channel-forming protein (VAC3_ORF2). Neither phage codes for tRNAs, and both have one predicted anti-CRISPR gene. Regarding phylogeny, VAC1 and VAC3 have over 97% genomic similarity with Casadabanvirus JBD69 (NCBI Reference Sequence: NC_030908.1), as determined by the taxmyPHAGE taxonomy tool^27^. It is important to note that both phages have ORFs annotated as integrases (VAC1_ORF52 and VAC3_ORF53), meaning that despite being good models in this study, their therapeutical potential in clinics should be considered. However, no experimental evidence of lysogeny has been noted during the experiments shown here.

### Phage VAC3 is preferentially retained in the respiratory tract of mice

**Fig. 3:**
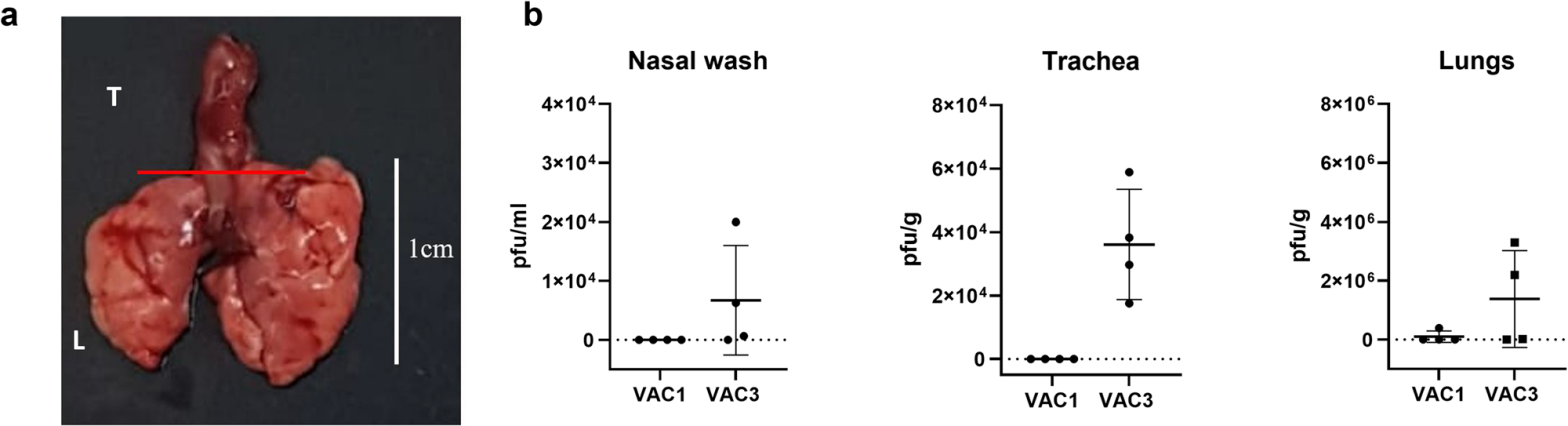
In vivo binding of phages VAC1 and VAC3 in different regions of the respiratory tract of C57BL/6 mice. **a** Representative figure showing the regions of the respiratory tract of mice used to quantify the bacteriophages. The respiratory tract was excised aseptically, and the trachea (T) and lungs (L) were used separately to quantify phages. The red line in A indicates the separation between the trachea and lungs. **b** Quantification of VAC1 (*n* = 4) and VAC3 (*n* = 4) in the nasal wash (pfu/mL), trachea (pfu/g), and lungs (pfu/g) of mice treated with these bacteriophages. The individual replicates are shown in (**b**), with the SD and mean marked in the graph. The limit of detection in **b** was 100 pfu/ml.

To verify the possibility of VAC1 and VAC3 adhering and persisting in the primary mucosal surface of the respiratory tract, C57BL/6 mice were treated via the nasal route with either phage. After 24 h, mice were euthanized, and the presence of phages in the trachea and lungs was checked by collecting tissue and quantifying the phages by titration. The material collected was divided between the trachea, lungs (Fig. 3a), and nasal wash. Phage titers indicate that phage VAC1 and VAC3 have a differential ability to persist in the different regions of the respiratory tract of the mice. As shown in Fig. 3b, phage VAC3 was recovered from the nasal wash and lungs of 75% and in the trachea of all treated mice. VAC1 was not detected in the nasal wash and trachea of the treated mice, being found in only one mouse lung in low titers. No phage plaques appeared when samples from the control group (mice not treated with phages) were tested, indicating the absence of detectable endogenous phages.

### Pretreatment with phage VAC3 improves survival and clinical signs when followed by a lethal dose of *P. aeruginosa*

**Fig. 4:**
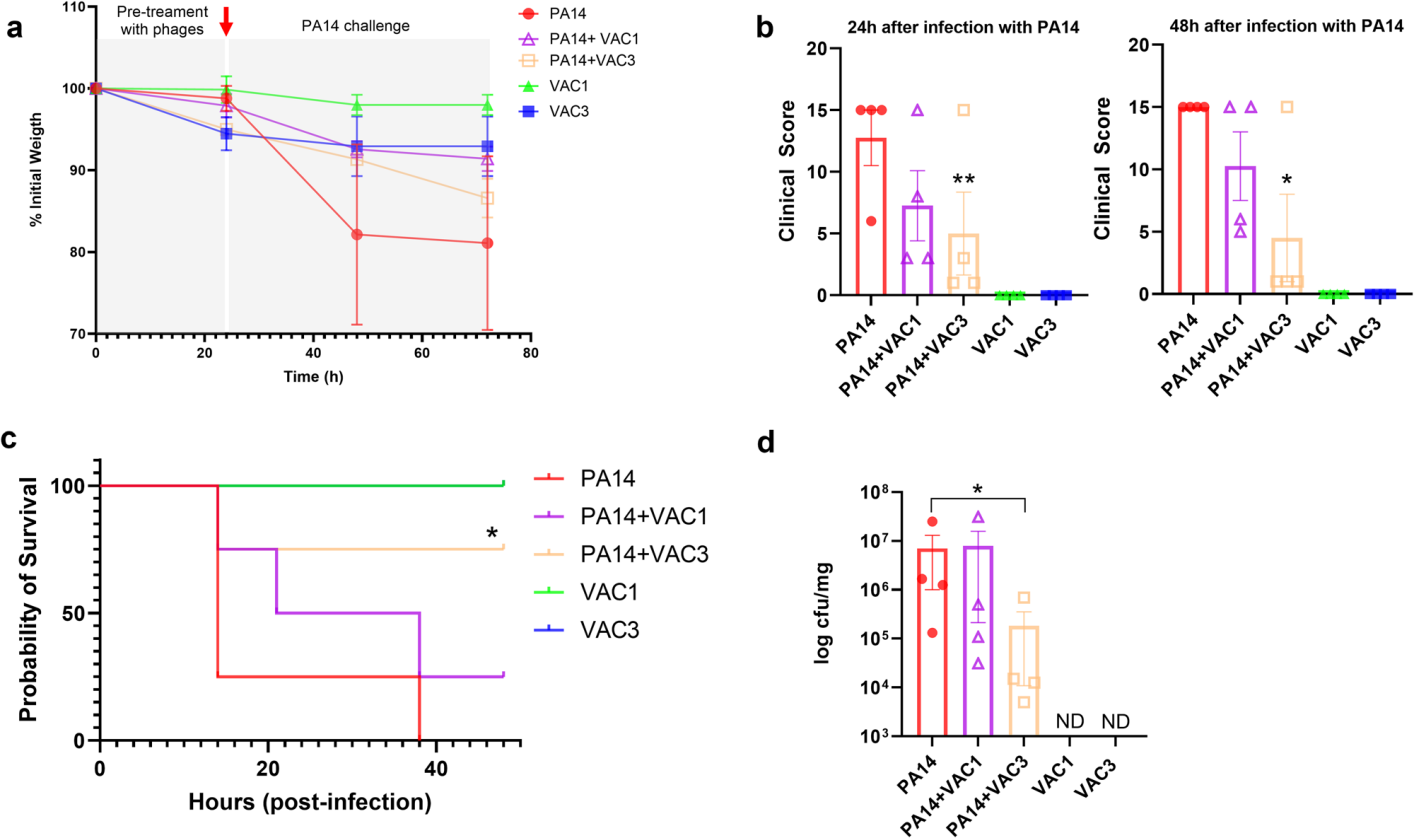
Pre-treatment of C57BL/6 mice with phage VAC3 protects C57BL/6 mice from lethal infection with *P. aeruginosa* PA14 strain. **a** Weight loss of mice during the first 24 h of pretreatment with phages and the following 48 h after infection with *P. aeruginosa* PA14 strain. The red arrow indicates the moment in which *P. aeruginosa* PA14 was administered to the mice. **b** Clinical sign scores in bacteriophages-treated mice after infection with *P. aeruginosa* PA14 strain 24- and 48-h post-infection with *P. aeruginosa*. Note that the 24 h and 48 h time points are counted after the administration of *P. aeruginosa* PA14. **c** Survival curve of phage-treated mice after infection with *P. aeruginosa* PA14 strain. The mice were monitored for 48 h after infection or until reaching a clinical score of 15 or death. The numbers of deaths and survivors were recorded. Note that the VAC1 and VAC3 curves are superposed (no death in both groups). **d** Quantification of colony forming units in the lungs of phage-treated mice after infection with *P. aeruginosa* PA14 strain. ND not detected. The graph shows the average and SD of each group comprised of four mice. The individual replicates plus SD are shown in (**b**) and (**d**). **Significance of *p* < 0.01 compared to PA14 group. *Significance of *p* < 0.05 compared to PA14 group. All groups had 4 mice (*n* = 4). The results were analyzed using Kruskal–Wallis non-parametric ANOVA for (**a**, **b** and **d**). Survival curves **c** were analyzed using Log-rank (Mantel–Cox) test.

After verifying the persistence of phage VAC3 along the respiratory system of mice, we checked whether pre-exposure with our phages could protect C57BL/6 mice. Therefore, mice pretreated via the nasal route with phages were infected 24 h after the phage treatment with a lethal dose of *P. aeruginosa* PA14, using the same route. During the course of infection, clinical parameters were followed. No significative difference was seen regarding the weight of mice in the different tested conditions (Fig. 4a), despite a drop in weight for the mice infected with the bacterium without phage pretreatment. However, mice in the groups pretreated with phages and then infected had fewer clinical signs compared to the non-pretreated mice, with a stronger reduction seen for the VAC3 pretreated group (p = 0.0025 in 24 h and *p* = 0.0147 in 48 h) (Fig. 4b). Both groups pretreated with phages had a higher survival rate when compared to the group not treated with phages (Fig. 4c). Of note, survival was higher and significant for the VAC3 pretreated group when compared to the non-treated group (*p* = 0.0122). One mouse survived the lethal dose of *P. aeruginosa* in the VAC1 pretreated group, while three survived in the VAC3 pretreated group. In addition, none of the mice treated with phages alone (no *P. aeruginosa* challenge) had recorded clinical symptoms or mortality, demonstrating that treatment with phages did not cause significant systemic changes. The quantification of bacteria in the lungs of mice shows that only treatment with phage VAC3 was able to reduce the number of colony-forming units (CFU) per milligram of tissue compared to the group of infected mice (*p* = 0.047) (Fig. 4d).

### Histopathology confirms the efficacy of VAC3 in diminishing *P. aeruginosa* damage

**Fig. 5:**
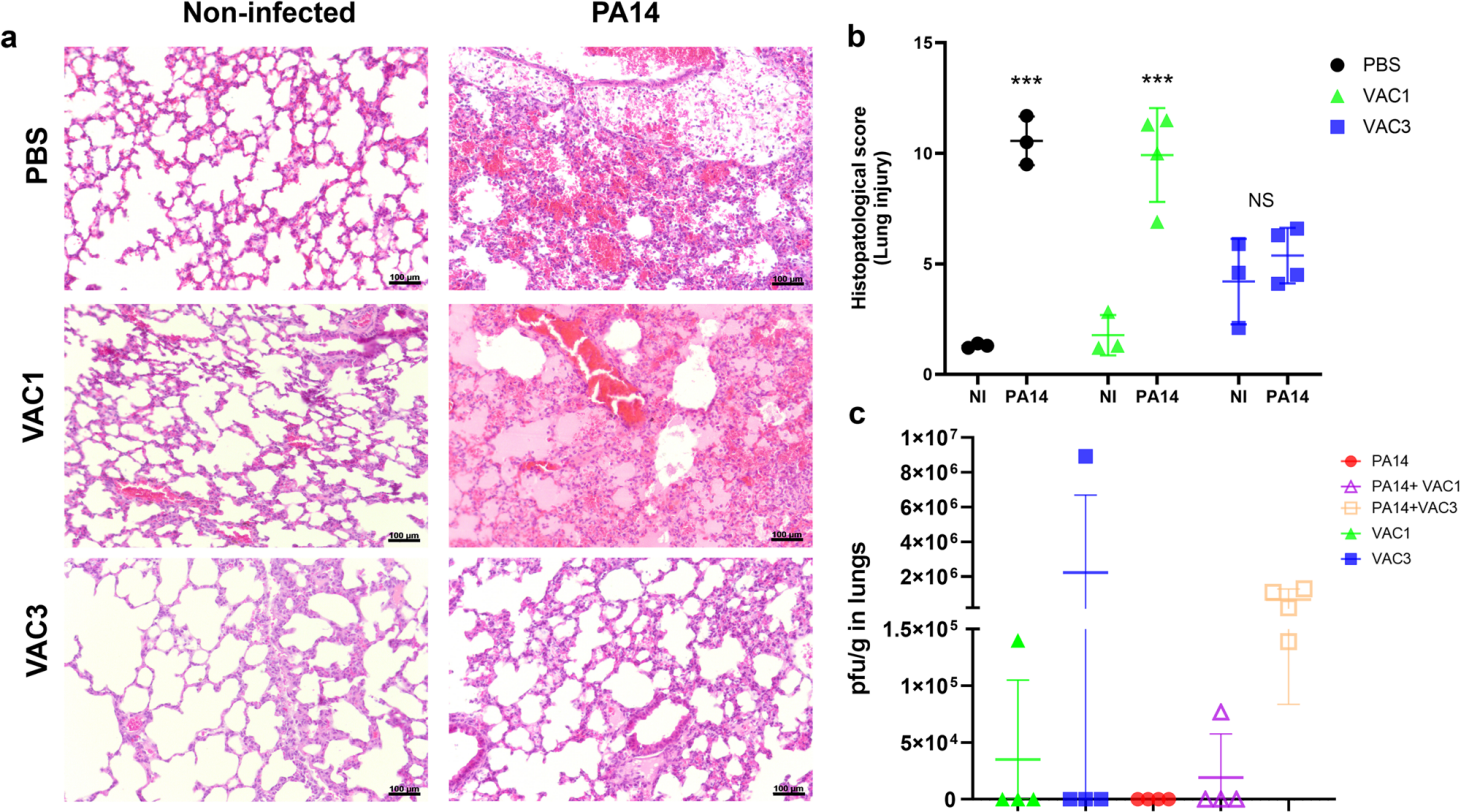
Histopathological changes in lungs of mice pre-treated with phages and challenged with *P. aeruginosa* PA14 strain. **a** Histopathological analysis of the lungs of C57BL/6 mice that were pre-treated with bacteriophages VAC1 and VAC3 before *P. aeruginosa* infection. Representative photomicrographs of the lung region in a 20× objective. Staining with hematoxylin and eosin (HE); magnification at 20×; scale bar: 100 μm. **b** Histopathology scores of lungs from C57BL/6 mice pre-treated with bacteriophages VAC1 and VAC3 before *P. aeruginosa* infection. **c** Quantification of VAC1 and VAC3 by titration in the lungs (pfu/g) of mice treated with both phages. NI is non-infected with *P. aeruginosa*. The individual replicates plus SD are shown in (**b**) and (**c**). *** Significance of *p* < 0.0001 compared to the non-infected group. The results were analyzed using an unpaired *t*-student test.

Biological material collected from the infection experiment shown in Fig. 4 was used for histopathology and *P. aeruginosa* colony counting. Histopathological analysis of lung sections showed areas of intense inflammation in all groups of mice infected with PA14 when compared to non-infected ones. However, lung injury was more intense in the PBS and VAC1 pretreated group compared to the VAC3 pretreated group (*p* = 0.0003 and *p* = 0.0018, respectively) (Fig. 5a, b). Infection with PA14 induced a greater loss of the alveolar architecture in the lung in all groups, with diminished effect in VAC3-treated mice. Thickening of the alveolar septum, foci of vascular congestion, an intense interstitial inflammatory infiltrate, and the presence of exudates in the PBS and VAC1 treated group after infection were observed. In contrast, VAC3 pretreated mice showed a similar lung architecture to non-infected mice (Fig. 5a). The lung injury histopathological score was similar between PBS and VAC1 groups after infection with PA14 (Fig. 5b). In contrast, mice treated with VAC3 showed a reduction of all inflammatory parameters after infection demonstrating a lower interstitial inflammatory infiltrate, little alveolar septum thickening and some few vascular congestion (Fig. 5a, b). The presence of phages in the lungs of infected mice was also determined. Phage titration from the lung indicated that only one mouse from VAC1 and one from VAC3 treated groups (not infected with *P. aeruginosa*) were positive for phage detection after 48 h of phage administration dose (Fig. 5c). When the same analysis was done in mice that were pretreated with phages and then infected with *P. aeruginosa*, phage VAC1 was detected in only one individual while all mice were positive for VAC3 phage (Fig. 5c).

## Discussion

*P. aeruginosa* is an opportunistic pathogen responsible for a wide range of acute and chronic infections with significant levels of morbidity and mortality. This bacterium is frequently found infecting patients with cystic fibrosis, cancer, severe burns, and also immunocompromised patients^10,11,28–30^. A complicating factor of the infection by *P. aeruginosa* is the presence of MDR and XDR isolates, which in turn compromise the antimicrobial therapy and increase the mortality and morbidity rates of the populations affected by this infection^31,32^. In vitro and in vivo studies have already demonstrated that bacteriophages are promising agents in the elimination or inhibition of *P. aeruginosa* MDR when used with a curative approach^7,12,13,21,30,33–42^.

A factor to be considered in the study of phages is their interaction with the environment, especially with the mucosal surfaces of various animals. These surfaces are habitats for microorganisms, including bacteriophages^19,22–25^. The BAM model suggests that phages would concentrate on the mucosal surfaces through weak interactions with mucins, creating a ubiquitous non-host-derived immunity against bacterial invaders during the mucus colonization process^19^. Additionally, some bacteria were already shown to have increased susceptibility to mucosal-adapted phages when exposed to mucin. This has been shown directly for *Flavobacterium columnare*, *Aeromonas sp*, *Streptococcus mutans*, *A. veronii* and *P. aeruginosa* phages^19,20,26,43^ and indirectly for *Clostridioides difficile* and *E. coli* phages^44,45^. This points out that likely phages associated with mucosal layers are effective predators of bacteria in their mucosal-invasive physiological state in nature. Phage therapy based on phages that bind to mucus has the potential to act as a preventive approach to protect the mucosa against infections that affect these sites^19^. The impact of phage-mucosa dynamics for phage-based prophylaxis of bacterial infections has already been shown in vivo for three distinct bacterial pathogens: *F. columnare*^19^*, A. veronii*^20^, and *E. coli*^21^.

In this work, we isolated two *P. aeruginosa* PA14 infecting phages from environmental water samples collected in Brazil. Despite being genetically and morphologically similar to each other, phage VAC3 was more efficient in proliferating in mucin-exposed *P. aeruginosa* in vitro, whereas VAC1 was not. Differential response of similar phages to mucin was also recently recorded in a different collection of *P. aeruginosa* phages from the Eliava Institute in Georgia^26^. When tested in vivo, VAC3 was preferentially held in the respiratory system of mice, while VAC1 was not. VAC3 retention was higher in the trachea, an organ that has areas containing large clouds of mucus along the respiratory apparatus^46^. The absence of VAC1 phage along the trachea and nasal region of the treated mice could be associated with the passage of the virus to the lungs without adherence to areas containing primary mucus. The mechanism behind VAC3 attachment to the upper respiratory tract is still not understood, and it is important to note that this phage lacks the canonical HOC protein shown to mediate phage-mucin interactions in T4 phage^14^. Differential stability between VAC1 and VAC3 is likely not an issue, given that both phages are similar and survived equally under different temperature and pH treatments. Pretreatment of mice with VAC3 resulted in a higher survival rate, fewer clinical signs, and lower bacterial counts in the lungs of the mice. This protection could be associated with the fact that the presence of VAC3 phages in the nasal cavity and trachea before infection could favor the infection of bacteria by this phage at these two anatomical sites. This could, at the same time, reduce live *P. aeruginosa* cells in the lungs and increase the production of a new viral progeny of VAC3 phage in the infected tissues. The lower degree of tissue injury and inflammatory parameters in mice pre-treated with VAC3 phages in comparison to VAC1, together with the high detection rate of VAC3 phage titers in the lungs of all mice after infection, strengthen this hypothesis.

So far, the rational prophylactic use of phages based on mucosal interactions in vivo has been shown in two different fish disease models^19,26^ and against *E. coli* in BALB/c mice^21^. To our knowledge, this is the first time the BAM model dynamics were applied to prevent *P. aeruginosa* infections in vivo, pointing out to the potential relevance of such an approach against ESKAPE pathogens. The success of the use of phage therapy against *P. aeruginosa* in vitro and in vivo models is already well established^30,40–42^. The main difference between those studies and our study is the use and validation of preventive phage therapy based on a phage that remains attached to the upper respiratory tract and replicates more efficiently in the presence of mucin. This approach could be important to prevent *P. aeruginosa* infections in patients with CF. CF is a genetic-based disease, characterized by higher concentrations of mucins in the lungs of affected individuals. This scenario contributes to the establishment of a chronic inflammatory response in the lungs and also airway obstruction^47^. Together with this hypersecretion mucus phenotype, there is a chronic bacterial infection that could, in turn, exacerbate lung inflammation^48^. One of the major bacterial pathogens infecting CF patients is *P. aeruginosa*^49^. Therefore, the high frequency of antibiotic consumption by CF patients contributes to an increased rate of MDR pathogens in the lungs, including *P. aeruginosa*^50^. A multi-center clinical trial showed that phage therapy in CF patients infected with *P. aeruginosa* showed that the phage cocktail was well tolerated and also reduced bacterial load in the lungs of participants^51^.

Besides a high degree of identity, the VAC3 phage genome is 1062 base pairs longer than the VAC1 phage genome. Pangenome analysis showed that VAC1 has one unique ORF (VTF3S_VAR67 Intermediate transcription factor 3 small subunit). In general, Viral Intermediate transcription factors (VIF) are proteins that can act with host RNA polymerase to initiate transcription from viral intermediate gene promoters or inhibit host cell transcription^52^. VAC3 genome has two unique ORFs, a LysR substrate-binding domain-containing protein and a Nucleoside-specific channel-forming protein tsx. The LysR substrate-binding domain-containing protein is a transcription factor widely expressed in bacteria. When a LysR protein recognizes a specific substrate, there are conformational changes that modulate the protein’s DNA-binding affinity and regulatory activity^53^. The other unique ORF in VAC3 codifies a potential nucleoside-specific channel-forming protein tsx. This protein is a porin expressed in the outer membrane of some Gram-negative bacteria and is described as a substrate-specific channel for nucleosides and deoxynucleosides. It also could act as a receptor for colicin K and bacteriophage T6^54,55^. It was also demonstrated that mutations in the Tsx porin gene result in bacterial resistance to phage infection^56,57^. Therefore, the presence of these ORFs does not correlate directly with the differential mucosal retention of VAC1 and VAC3, since these ORFs are not identified as structural proteins. However, we could not exclude that these ORFs could be indirectly related to mucin or mucus response in phage-host interaction. VTF3S_VAR67 Intermediate transcription factor 3 small subunit and LysR substrate-binding domain-containing protein are involved in transcription regulation, and the activity of these transcription factors could be modulated by mucin exposure of the host. Additionally, the nucleoside-specific channel-forming protein tsx expressed by VAC3 could positively influence the infection of *P. aeruginosa* by this phage since once this protein channel is expressed in the outer membrane of infected bacteria, nucleoside transport across the bacterial is facilitated, and therefore precursors for viral nucleic acid synthesis. Future studies should be done to verify the real role of these ORFs in the differential mucin-binding phenotype, if any.

In conclusion, here we show the differential ability of two newly isolated *P. aeruginosa* phages to respond to mucin, persist in mucus-producing tissues, and protect mice against a lethal dose of the bacterium. Phage therapy based on mucosal-adapted phages has a protective potential against infections that affect mucosal surfaces, as the administration of these phages preventively could prevent bacterial colonization. Mucosal-adapted phages could be used for the development of preventive technologies (e.g., nasal sprays, sanitizing solutions for environmental use, or use in surgical equipment) that could act as preventive methods against infection in people linked to the hospital environment, enabling the protection of patients at risk as well as the protection of visitors/companions and health professionals.

## Methods

### *Pseudomonas aeruginosa*

*P. aeruginosa* strain PA14 was cultured in lysogeny broth (LB)^58^ at 37 °C and 150 rpm. In some experiments, cultures were made in LB supplemented with 0.1% of mucin (w/v). Mucin stocks were prepared by the autoclavation of a 2% mucin solution in water. The mucin used was purified porcine mucin (Sigma), shown previously to be a relevant model for studying phage–mucin interactions in the concentration used^19,26^.

### Bacteriophage isolation and propagation

Bacteriophages that infect the *P. aeruginosa* PA14 strain were isolated from environmental water samples using the enrichment method and a double-layer agar overlay^58^. Environmental water samples were collected from two cities in southeast Brazil (Alfenas, Minas Gerais State—21° 26′ 0″ South, 45° 57′ 0″ West and Mococa, São Paulo State- 21° 28′ 0″ South, 47° 1′ 0″ West). Samples were filtered through 0.45 μm pore size filters, and 3 mL of the filtrates were mixed with 1 mL of 5× LB and 1 mL of a 48-h culture of *P. aeruginosa* for preparing the enrichment cultures. After 24 h incubation at 37 °C and 150 rpm, the cultures were centrifuged at 4000*g* for 30 min at 25 °C, and the supernatants were filtered through a 0.45 μm pore size filter. Dilutions (10^−1^ to 10^−5^) of enriched samples were plated by the overlay agar method containing PA14 strain. Plaques with differential morphology were picked and suspended in TM buffer (50 mM Tris–HCl, 10 mM MgCl_2_, pH 7.4). This suspension was further 10-fold serially diluted and used for plaque re-isolation. To ensure purity, each plaque was re-isolated three times before the experiments.

### Transmission electron microscopy

Ten microlitres of a phage lysate solution (1 × 10^10^ pfu/mL) were added on a carbon film over the copper grid for negative staining with 2% phosphotungstic acid (pH 6.7). The grids were imaged in a Tecnai G2-12—FEI Spirit Biotwin 120 keV transmission electron microscope.

### Effect of pH and temperature on phage stability

One hundred microliters of a phage suspension (1 × 10^11^ pfu/mL) was added to tubes containing 0.9 mL of sterile buffered saline with pH levels adjusted between 3 and 9 using 0.1 M HCl or NaOH. The phages were then incubated at 37 °C for 1 h, followed by the determination of phage titers using the double-layer agar method. Similarly, 100 μl of phage suspension was added to tubes containing 0.9 mL of sterile buffered saline and allowed to incubate in a heat block ranging from 30 °C to 60 °C for 1 h. Phage titers were determined using the double-layer agar method after the incubation period.

### Sequencing, assembly, and annotation

Phage DNA was extracted from 1 mL of high-titer phage lysates (1 × 10^10^ pfu/mL) utilizing the ZnCl_2_ method as described by Santos et al.^59^. Following filtration, DNase (10 µg/mL) (Thermo Fischer) and RNase (10 µg/mL) (Thermo Fischer) were added to the lysates and incubated at 37 °C for 1 h. Subsequently, the particles were concentrated using 0.2 M filtered ZnCl_2_ (Sigma) and treated with 1 mg/mL of Proteinase K. Finally, DNA extraction was performed using a DNA extraction kit (NucleoSpin Blood Kit, Macherey-Nagel).

For DNA Library preparation, the Ready-To-Go™ GenomiPhi™ V3 DNA system (Cytiva®) was used following the manufacturer’s instructions to amplify DNA. The concentration of each sample was determined using the Qubit™ dsDNA HS Assay Kit (Invitrogen®), with initial concentrations of 4.73 ng/μL for VAC1 and 8.3 ng/μL for VAC3 genomic DNA. Each sample was diluted in nuclease-free water to achieve an input of approximately 10 ng in a 10 μL volume. After amplification, the concentrations were 357 ng/μL for VAC1 and 378 ng/μL for VAC3, respectively. The NGS library was prepared using the COVIDSeq kit (Illumina®), following the manufacturer’s procedural guide. The process began with the Tagment PCR Amplicons step, using 357 ng for VAC1 and 378 ng for VAC3 in a total volume of 20 μL per sample, adjusted with nuclease-free water as needed. The final library concentrations were 29.6 ng/μL for VAC1 and 26.9 ng/μL for VAC3, with an average fragment size of 434 bp for both. Sequencing was performed on a MiSeq system using a 600-cycle V3 flow cell.

The genomes were assembled de novo using Spades 3.12 software with the default parameters^60,61^. Synteny analysis was performed on PhageScope^62^. Open reading frames (ORFs) were predicted using Prodigal^63^. All of the predicted ORFs were annotated manually using BLASTp (considering a cutoff of <10^−3^), HHpred (considering only hits with probabilities greater than 80% and E value greater than or equal to 1 found in the PDB_mmCIF70_8_Mar, SCOPe70_2.08 and UniProt-SwissProt-viral70_3Nov_2021 databases) and InterPro, during March of 2024^64–66^. The pangenome was constructed using the OrthoFinder 2.5.4 software with an MCL inflation parameter equal to 4^67^. To visualize the constructed pangenome, a network was built using Gephi^68^. The layout was generated using a force-based algorithm.

For phylogenetic analysis, amino acid sequences of homologous major capsid proteins were obtained from the National Center for Biotechnology Information database using the BLASTp (<10^−5^) and aligned using the MUSCLE algorithm. After aligning these sequences, the best-fit substitution models were selected by the ModelFinder algorithm implemented in IQtree^69^. The phylogenetic tree was reconstructed using IQtree software (version 1.6.12)^70^, using the maximum-likelihood method and 1000 bootstrap replicates. Finally, the phylogenetic tree was visualized and edited using iTOL^71^.

### Phage infections

To analyze the effect of our phages in liquid cultures of *P. aeruginosa* PA14, overnight cell cultures were adjusted to 2 × 10^8^ cells/mL in sterile saline solution (0.9% NaCl). A 100 μl of cell suspension was inoculated in 5 mL of LB medium followed by incubation at 37 °C with regular shaking (120 rpm). The culture was infected with phages at the multiplicity of infection (MOI) of 0.01. After the desired times of incubation, samples were taken to quantify the number of cells by spectrophotometry at an optical density of 600 nm. To evaluate the influence of mucin supplementation in phage multiplication, overnight cultures were diluted to 1:10,000 and inoculated to LB supplemented with 0.1% mucin. Control cultures consisted of an LB without mucin. All samples were incubated at 37 °C for 4 h and after they were infected with phages at MOI of 0.01 After 4, 24, and 48 h of infection, samples were collected (100 µl each), mixed with 10 μl of chloroform, and titrated as described above.

### In vivo binding of phages throughout the respiratory system of mice

To verify the ability of the phages to persist in the respiratory system, mice anesthetized intraperitoneally with xylazine (10 mg/kg) were treated with 10 μL of bacteriophages diluted in SM buffer (200 mM NaCl2 10 mM MgSO_4_ 50 mM Tris-HCl, pH 7.5) via nasal route in each nostril (2 × 10^8^ PFU/nostril). After 24 h, all mice were euthanized through a lethal dose of anesthetics (ketamine at 300 mg/kg and xylazine at 30 mg/kg). The respiratory system of each individual was externalized and divided into tracheal and pulmonary portions. The organs (trachea and lungs) were weighted and homogenized in 500 μl of sterile PBS. The homogenates were centrifuged at 10,000 rpm for 5 min, and the supernatant was used for bacteriophage titration. The nasal wash was collected using sterile PBS as described^72^. Briefly, after extracting the respiratory system, a syringe was inserted into the larynx region, and a sterile 1.5 mL tube was inserted in the nostril. Lavages were performed with 500 μl of sterile PBS, which was flushed through the larynx and collected through the nares in a 1.5 mL microtube. The collected sample was centrifuged as described above, and a supernatant was used for bacteriophage titration.

### Prophylactic effect of phages against a *P. aeruginosa* challenge in vivo

Mice were treated with phages as described in the topic above. Twenty-four hours after the phage treatment, mice were anesthetized with xylazine (10 mg/kg) and challenged with 10 μL of *P. aeruginosa* PA14 in LB medium via the nasal route in each nostril (4 × 10^7^ colony forming units/nostril). Mice were monitored every 8 h post infection, and survival was assessed until 48 h after infection. Clinical parameters were evaluated using the clinical parameters described by Cigana et al.^73^. Based on these parameters, a final total score of up to 15 points was calculated as a global indicator of the clinical symptoms (Supplementary Table 1). The right lungs of mice that died or reached an endpoint clinical score of 15 in this period were collected to quantify the number of colony-forming units (CFU) of *P. aeruginosa* per milligram of tissue. The left lungs of mice were preserved for histopathological analysis. For mice that survived beyond 48 h, euthanasia was performed through a lethal dose of anesthetics, and both right and left lungs were acquired for analysis, following the previously outlined procedure.

### Lung microbiological and Histological analysis

The right lung from each mouse was excised and homogenized in 1 mL of sterile PBS. Subsequently, 100 μl of serially diluted lung homogenate samples were inoculated to LB agar. The culture plates underwent incubation at 37 °C, and the plates were examined for the presence of *P. aeruginosa* colonies after 24 h. For phage quantification, 100 μl of lung homogenates were mixed with 10 μl of chloroform and titrated as described above. The left lung from each specimen was preserved using 10% formaldehyde in PBS at room temperature. Tissues were submitted to dehydration in ethanol and embedded in paraffin. Semi-serial sections, measuring 5 μm in thickness, were stained with hematoxylin and eosin, and tissue slides were examined utilizing a Carl Zeiss Axio ScopeA1 microscope equipped with a Canon G10 Power Shot digital microscope camera. For both histopathological and morphometric examinations, 10 randomly selected histological images were acquired for analysis. The sections were evaluated in a double-blind way concerning lung injury was assessed using a modified scoring system, which comprised four categories: edema, hemorrhage, leukocyte infiltration, and vasocongestion. Each category was assigned a score ranging from 0 to 3. The total lung injury score was determined by adding together the scores from each category. Thus, the maximum possible score for all histological parameters combined was 12^74^. Additionally, the loss of alveolar architecture in the lungs of mice was also measured. In this case, this parameter was graded from 0 (normal alveolar architecture) to 4 (severe loss of alveolar architecture).

### Statistics and reproducibility

Graphs and statistical analysis were made using GraphPad Prism version 9.3.1 (GraphPad Software, San Diego, California, USA). Conditions were tested in triplicates, and unpaired *t*-tests were used to compare controls and tested conditions whenever necessary. Some results were analyzed using Kruskal–Wallis non-parametric ANOVA with two or more groups present. Survival curves were analyzed using Log-rank (Mantel–Cox) test. A *p* value below 0.05 was considered to be significative and the precise *p* values are indicated in the text or in the figures. Group size was calculated to be four mice in each tested condition the second equation from Dell et al.^75^, as recommended by the local regulations. The figure legends contain specific information regarding replicates for each experiment. The raw data used for preparing the graphs in this paper are provided in the Supplementary Data set 2.

### Reporting summary

Further information on research design is available in the Nature Portfolio Reporting Summary linked to this article.

## Supplementary information

Supplementary material

Description of Additional Supplementary Files

Suplemmentary data 1

Suplemmentary data 2

Reporting Summary

## Data Availability

Source data used for making the graphs can be found in Supplementary Data set 2. The phages used in this study are not available in biological collections but could be shared after consideration of local regulations and MTA signing with interested parties. Phage genomes are deposited in Genbank under the following accession numbers PQ466444 (VAC1) and PQ466445 (VAC3).
