# Supplementary material for: Mucosal-adapted bacteriophages as a preventive strategy for a lethal Pseudomonas aeruginosa challenge in mice
Source: Commun Biol. 2025 Jan 6;8:13. doi: 10.1038/s42003-024-07269-0 (PMC11704353; doi:10.1038/s42003-024-07269-0)
Supplement: Supplementary file 1 — Supplementary material [file 42003_2024_7269_MOESM1_ESM.pdf]

Supplementary material

Mucosal-adapted bacteriophages as a preventive strategy for a lethal *Pseudomonas aeruginosa* challenge in mice

Luiz Felipe Leomil Coelho[1], Mateus de Souza Terceti[1], Sergio Pereira Lima Neto[1], Raíne Piva Amaral[1], Ana Luisa Cauvila do Santos[1], William Permagnani Gozzi[1], Bianca Andrade de Carvalho[1], Gustavo Aparecido da Cunha[1], Maria Fernanda Romboli Durante[1], Lais Sanchietta[1], Giovana Soares Marangoni[1], Matheus Luca Carotta Gabriel[1], Luiz Cosme Cotta Malaquias[1], Eliana Leonor Hurtado Celis[2], Giovanna de Souza Apolinário[2], João Pessoa Araujo Junior[2], Carine Ervolino de Oliveira[3], Victoria Fulgencio Queiroz[4], Gabriel Magno de Freitas Almeida[5]*

[1] Vaccine Laboratory, Department of Microbiology and Immunology, Institute of Biomedical Sciences, Federal University of Alfenas, Alfenas, Brazil.

[2] Institute of Biotechnology, Paulista State University (UNESP), Sao Paulo, Brazil.

[3] Institute of Biomedical Sciences, Department of Pathology and Parasitology, Federal University of Alfenas, Alfenas, Brazil.

[4] Federal University of Minas Gerais, Institute of Biological Sciences, Department of Microbiology, Belo Horizonte, Minas Gerais, Brazil

[5] The Norwegian College of Fishery Science, Faculty of Biosciences, Fisheries and Economics, UiT - The Arctic University of Norway, Tromsø, Norway.

*Corresponding author. Email address: gabriel.d.almeida@uit.no

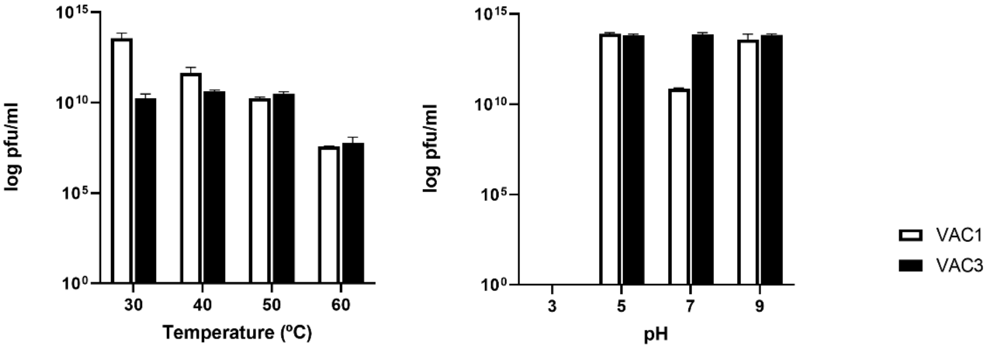

Supplementary figure 1: Resistance test of phages VAC1 and VAC3 to different temperatures and pH. One hundred microliters of phage suspension ($1x10^{11}$ pfu/mL) was added to tubes containing 0.9 mL of sterile buffered saline with pH levels adjusted between 3 and 9. Similarly, 100 µl of phage suspension was added to tubes containing 0.9 mL of sterile buffered saline and incubated in a heat block ranging from 30°C to 60°C. The phages were then incubated for 1 hour followed by the determination of phage titers using the double-layer agar method.

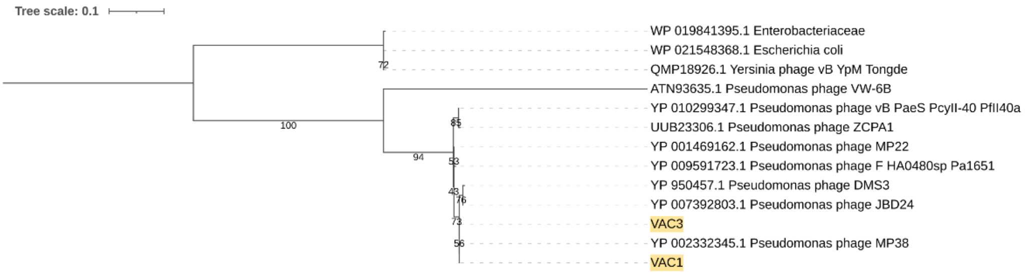

Supplementary figure 2: Phylogenetic tree of the bacteriophages VAC1 and VAC3. Maximum likelihood based phylogenetic tree of the MCP gene constructed using IQ-TREE multicore version 2.2.0. The best-fit model was WAG+I chose according to BIC. The branch supports were computed by 1000 ultrafast bootstrap. The phylogenetic tree was visualized and edited using iTOL.

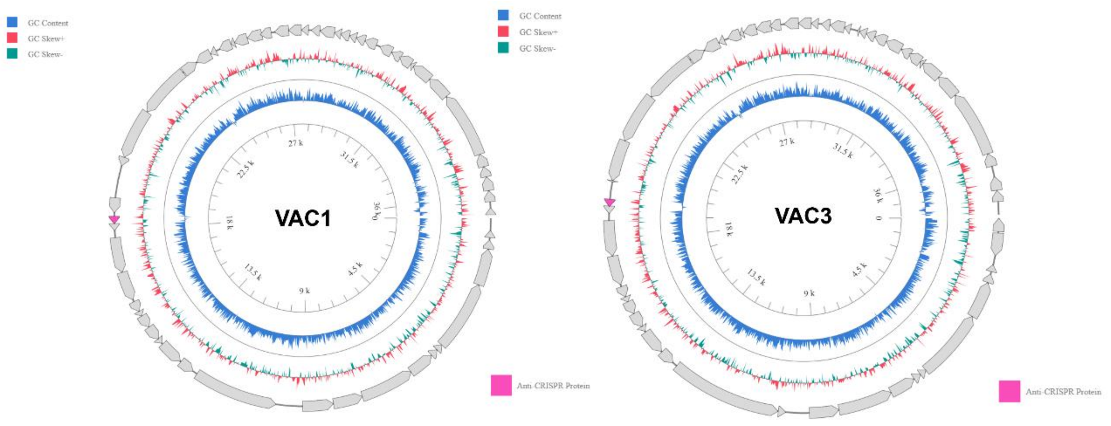

Supplementary figure 3: Circular genome map of VAC1 and VAC3 phages. Arrows in the outer circles indicate the predicted ORFs in their direction of transcription. The blue circle represents the GC content. The green/red circles represent the GC-skew calculated. Anti-CRISP proteins were represented in pink.

Supplementary Table 1. Clinical score evaluation parameters used. A score of 15 was used as criteria for endpoint.

**Clinical parameter**		**Score**
**Appearance**	Normal	0
	Lack of general cleanliness	1
	Nasal and ocular discharge	2
	Piloerection	3
	Painful posture	4
**Weight loss**	No loss	0
	< 5%	1
	5-10%	2
	10-25%	3
	> 25%	4
**Respiratory rate**	Normal	0
	> 10%	1
	> 30%	2
	> 50%	3
**Natural behavior**	Normal	0
	Minimal change	1
	loss of appetite	2
	Vocalizing	3
	Inactivity	4
	**TOTAL**	
