# Supplementary material for: Mucosal-adapted bacteriophages as a preventive strategy for a lethal Pseudomonas aeruginosa challenge in mice
Source: Commun Biol. 2025 Jan 6;8:13. doi: 10.1038/s42003-024-07269-0 (PMC11704353; doi:10.1038/s42003-024-07269-0)
Supplement: Supplementary file 2 — Description of Additional Supplementary Files [file 42003_2024_7269_MOESM2_ESM.pdf]

Description of Additional Supplementary Files

File name: Supplementary dataset 1

Description: ORF prediction and annotation of phages VAC1 and VAC3.

File name: Supplementary dataset 2

Description: Raw data used for preparing the graphs.
