# Supplementary material for: Mucosal-adapted bacteriophages as a preventive strategy for a lethal Pseudomonas aeruginosa challenge in mice
Source: Commun Biol. 2025 Jan 6;8:13. doi: 10.1038/s42003-024-07269-0 (PMC11704353; doi:10.1038/s42003-024-07269-0)
Supplement: Supplementary file 5 — Reporting Summary [file 42003_2024_7269_MOESM5_ESM.pdf]

nature portfolio

Corresponding author(s): Gabriel Magno de Freitas Almeida

Last updated by author(s): Oct 17, 2024

Reporting Summary

Nature Portfolio wishes to improve the reproducibility of the work that we publish. This form provides structure for consistency and transparency in reporting. For further information on Nature Portfolio policies, see our Editorial Policies and the Editorial Policy Checklist.

Statistics

For all statistical analyses, confirm that the following items are present in the figure legend, table legend, main text, or Methods section.

n/a	Confirmed	
☐	☒	The exact sample size (*n*) for each experimental group/condition, given as a discrete number and unit of measurement
☐	☒	A statement on whether measurements were taken from distinct samples or whether the same sample was measured repeatedly
☐	☒	The statistical test(s) used AND whether they are one- or two-sided<br>*Only common tests should be described solely by name; describe more complex techniques in the Methods section.*
☐	☒	A description of all covariates tested
☒	☐	A description of any assumptions or corrections, such as tests of normality and adjustment for multiple comparisons
☐	☒	A full description of the statistical parameters including central tendency (e.g. means) or other basic estimates (e.g. regression coefficient) AND variation (e.g. standard deviation) or associated estimates of uncertainty (e.g. confidence intervals)
☒	☐	For null hypothesis testing, the test statistic (e.g. *F*, *t*, *r*) with confidence intervals, effect sizes, degrees of freedom and *P* value noted<br>*Give P values as exact values whenever suitable.*
☒	☐	For Bayesian analysis, information on the choice of priors and Markov chain Monte Carlo settings
☒	☐	For hierarchical and complex designs, identification of the appropriate level for tests and full reporting of outcomes
☒	☐	Estimates of effect sizes (e.g. Cohen's *d*, Pearson's *r*), indicating how they were calculated

Our web collection on statistics for biologists contains articles on many of the points above.

Software and code

Policy information about availability of computer code

Data collection	NA
Data analysis	NA

For manuscripts utilizing custom algorithms or software that are central to the research but not yet described in published literature, software must be made available to editors and reviewers. We strongly encourage code deposition in a community repository (e.g. GitHub). See the Nature Portfolio guidelines for submitting code & software for further information.

Data

Policy information about availability of data

All manuscripts must include a data availability statement. This statement should provide the following information, where applicable:

- Accession codes, unique identifiers, or web links for publicly available datasets
- A description of any restrictions on data availability
- For clinical datasets or third party data, please ensure that the statement adheres to our policy

Source data used for making the graphs can be found in supplementary data set 2. The phages used in this study are not available in biological collections but could be shared after consideration of local regulations and MTA signing with interested parties. Phage genomes are deposited in Genbank under the following accession numbers: PQ466444 (VAC1) and PQ466445 (VAC3).

Human research participants

Policy information about studies involving human research participants and Sex and Gender in Research.

Reporting on sex and gender	NA
Population characteristics	NA
Recruitment	NA
Ethics oversight	NA

Note that full information on the approval of the study protocol must also be provided in the manuscript.

Field-specific reporting

Please select the one below that is the best fit for your research. If you are not sure, read the appropriate sections before making your selection.

☒ Life sciences ☐ Behavioural & social sciences ☐ Ecological, evolutionary & environmental sciences

For a reference copy of the document with all sections, see nature.com/documents/nr-reporting-summary-flat.pdf

Life sciences study design

All studies must disclose on these points even when the disclosure is negative.

Sample size	No sample size calculation was made. Experiments in vitro had at least three replicates and in vivo experiments used four mice per treatment group, as approved by the Ethics Committee. Group size of mice was calculated to be four mice in each tested condition the second equation from Dell et al 200275, as recommended by the local regulations.
Data exclusions	No data was excluded from the experiments.
Replication	Results were replicable.
Randomization	No randomization was needed.
Blinding	Blinding was not relevant to most of this study. However, to avoid observer bias in the scoring of histopathological samples, blinding was applied. This is explained in the methods section, as follows: "The sections were evaluated in a double-blind way concerning lung injury was assessed using a modified scoring system, which comprised four categories: edema, hemorrhage, leukocyte infiltration, and vasocongestion. Each category was assigned a score ranging from 0 to 3.".

Reporting for specific materials, systems and methods

We require information from authors about some types of materials, experimental systems and methods used in many studies. Here, indicate whether each material, system or method listed is relevant to your study. If you are not sure if a list item applies to your research, read the appropriate section before selecting a response.

Materials & experimental systems

n/a	Involved in the study	
☒	☐	Antibodies
☒	☐	Eukaryotic cell lines
☒	☐	Palaeontology and archaeology
☐	☒	Animals and other organisms
☒	☐	Clinical data
☒	☐	Dual use research of concern

Methods

n/a	Involved in the study	
☒	☐	ChIP-seq
☒	☐	Flow cytometry
☒	☐	MRI-based neuroimaging

Animals and other research organisms

Policy information about studies involving animals; ARRIVE guidelines recommended for reporting animal research, and Sex and Gender in Research

Laboratory animals	Eight-week-old female C57BL/6 mice.
Wild animals	This study did not involve wild animals.
Reporting on sex	Only female mice were used in this study.
Field-collected samples	Only water samples were collected. The samples were kept on cold prior to the phage isolation experiments described.
Ethics oversight	The Institutional Animal Care and Use Committee approved all animal experimental protocols (0041/2021) of the Federal University of Alfenas (Minas Gerais, Brazil). We have complied with all relevant ethical regulations for animal use. All eight-week-old female Mus musculus C57BL/6 mice used in this study were maintained with free access to food and water according to the local animal welfare regulations (protocol 0041/2021).

Note that full information on the approval of the study protocol must also be provided in the manuscript.
